# Rats use strategies to make object choices in spontaneous object recognition tasks

**DOI:** 10.1038/s41598-022-21537-1

**Published:** 2022-10-10

**Authors:** T. W. Ross, A. Easton

**Affiliations:** 1 Department of Psychology, Durham University, South Road, Durham, DH1 3LE UK; 2 Centre for Learning and Memory Processes, Durham University, Durham, UK

**Keywords:** Learning and memory, Behavioural methods

## Abstract

Rodent spontaneous object recognition (SOR) paradigms are widely used to study the mechanisms of complex memory in many laboratories. Due to the absence of explicit reinforcement in these tasks, there is an underlying assumption that object exploratory behaviour is ‘spontaneous’. However, rodents can strategise, readily adapting their behaviour depending on the current information available and prior predications formed from learning and memory. Here, using the object-place-context (episodic-like) recognition task and novel analytic methods relying on multiple trials within a single session, we demonstrate that rats use a context-based or recency-based object recognition strategy for the same types of trials, depending on task conditions. Exposure to occasional ambiguous conditions changed animals’ responses towards a recency-based preference. However, more salient and predictable conditions led to animals exploring objects on the basis of episodic novelty reliant on contextual information. The results have important implications for future research using SOR tasks, especially in the way experimenters design, analyse and interpret object recognition experiments in non-human animals.

## Introduction

**Figure 1:**
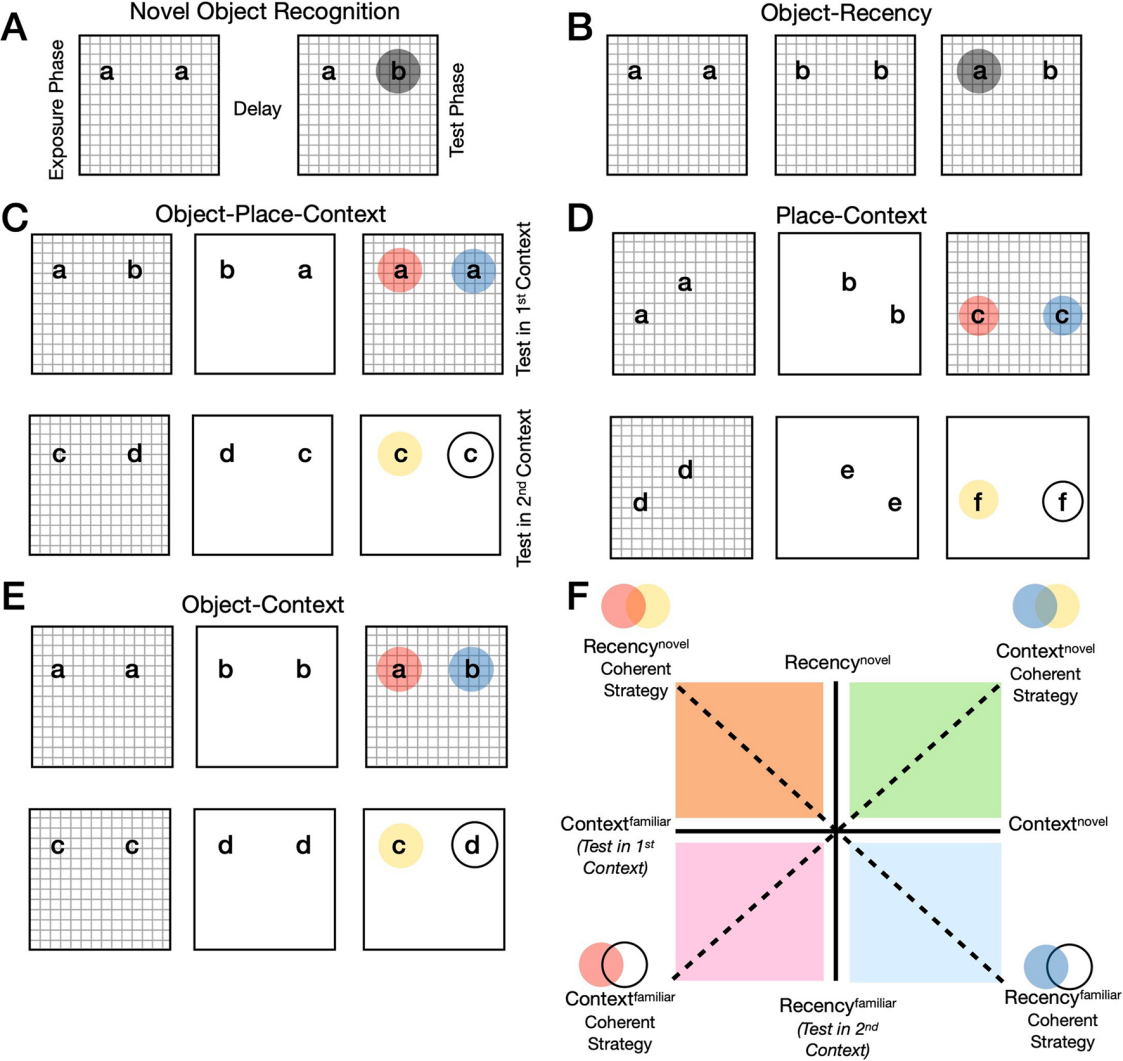
Schematics of various object recognition tasks with 2 objects. (**A**) Standard novel object recognition in the same environment with 1 exposure phase (left), a delay and a novel object present at the test phase (right). The highlighted grey circle denotes expected novelty-based discrimination. Lower case letters denote objects. (**B**) Object-recency task in the same environment with 2 exposure phases (left and middle) and the test phase (right). The highlighted grey circle denotes expected novelty-based discrimination on the object least recently seen. (**C**) Object-place-context task in two different environments (i.e., contexts) with 2 exposure phases (left and middle). Test phase can be made in the 1st context (upper right) or test can be made in the 2nd context (lower right). In test in the 1st context trials, the highlighted blue circle can denote novelty discrimination of integrated object-place-context association or the highlighted red circle can denote novelty discrimination of object in place recency (note that discrimination is ignorant to context, see **B**). In test in the 2nd context trials, the highlighted yellow circle can denote novelty discrimination of integrated object-place-context or novelty discrimination of object-place recency (they are overlapping). (**D**,**E**) Other 2 objects/exposure/contexts tasks which are susceptible to a context-based versus recency-based strategy where **F** is applicable. (**F**) Strategy scatterplot for 2 object/context/exposure object recognition tasks. Based on (**C**–**E**) one can average discrimination ratio scores separately for both for test in the 1st context trials and test in the 2nd context trials and plot them for each animal. The average context D2 for trials when test was made in the 1st context on the x-axis, and for trials when test was made in the 2nd context on the y-axis. Thus, one can form angular data and use directional statistics, which can enhance interpretive power if animals are behaving differently to chance. Context^novel^/Recency^novel^ denote exploration on the basis of object related novelty preference. Context^familiar^/Recency^familiar^ denote exploration on the basis of object related familiarity preference.

Spontaneous object recognition tasks are critical for allowing cross-species comparisons of complex recognition memory to enhance our mechanistic understanding^1–6^. In the standard version of novel object recognition, a single trial consists of an exposure phase and test phase^7^ (Fig. 1A). Rodents typically display successful object recognition memory via novelty preference in this version (i.e., exploring the novel object more than the familiar object, and to a greater extent than chance)^7–9^. However, successful memory expression can also be shown via preference for the familiar object in these tasks^10–12^. Memory is simply determined by preference of one object over another on the basis of past experience. The direction of that preference (for the novel or familiar object) can be driven by external factors, such as anxiety^1,12^.

The underlying presumption of SOR tasks is that they are training-free paradigms^13^, where the exploration behaviour at the test phase is an unconditioned preference^1^ and hence the exploratory preference for an object is spontaneous. In other words, as there is no explicit reinforcement used by experimenters, a strategy should not be learnt in these paradigms compared to other training-based (reinforced) tasks^13^. However, the neural mechanisms that give rise to novelty detection and novelty-seeking motivation^14–16^ allows for learning through the identification of novelty (which is inherent in SOR tasks) and this in itself can be behaviourally reinforcing^17^.

The mammalian brain also segments experience into discrete events^18,19^, and animals can formulate predictions of what to expect in certain situations based on prior knowledge learnt from their memory of experiences in past events^19–21^. This aims to minimise surprise and so when error of predictions is experienced, animals can flexibly update and guide their future behaviour^18,22^. Therefore, such evidence provides a strong basis for the possibility that rodents may be making strategic choices in SOR tasks.

Recent development of a continual trials approach to SOR tasks^3,9,23–25^ (i.e., running multiple trials within a single session, opposed to one trial a day), means that we can begin to obtain consistent behavioural choices from a single animal over several trials. This aims to reduce the number of total animals, whilst maintaining sufficient statistical power^23^ and has offered a novel opportunity to explore whether rodents are behaving coherently using a certain strategy over another.

The object-recency or temporal order recognition task^26,27^ (see Fig. 1B) uses two exposure phases before test in a single environment, which constitutes one trial. At test, rodents preferentially explore the novelty of the object seen least recently in this task^26–29^. On the other hand, context-based SOR tasks^30–32^ (Fig. 1C–E), which usually use distinct environments as contextual information, also use two exposure phases and a test as a single trial. Rodents can preferentially explore novel objects in context or novel objects in place and context more so than chance at the test phases^30–32^.

Recognition of simultaneous object-place-context (OPC) integrations fulfils the requirement of a behavioural definition of episodic memory^4,33^. Thus, if animals were using a novelty driven episodic strategy (based on context) in the OPC recognition task^30^, we can expect them to explore the novel object in place and context integration when test is made in the 1st context (the blue circle in Fig. 1C). However in the same OPC task, animals could also be exploring the novelty of the object least recently seen when test is made in the 1st context and this choice would be ignorant to contextual information (the red circle in Fig. 1C). Moreover, it is ambiguous whether animals are using a context-based or recency-based strategy in test in the 2nd context trials, as both predict the same object choice (the yellow circle in Fig. 1C). Therefore, when test phases of trials are made in the 1st context, object-recency memory and object-context memory are in opposition, whereas when test is made in the 2nd context they are overlapping^34^. Firstly, this suggests that it is important to use both types of trials to determine coherent behaviour (see Fig. 1F) and secondly it is possible that different recognition strategies can exist in the same task.

Here, we use a novel unexpected OPC task with a continual trials approach, and find that rats robustly change their recognition guided behaviour to a recency-based strategy expressed via familiarity preference, after experiencing unexpected ambiguous events. Yet, they could also exhibit a novelty driven episodic strategy in the same task, suggesting that strategy implementation was dependent on task conditions and hence not spontaneous.

## Results

### Rats change their behaviour to a familiarity driven recency-based recognition strategy after probe trials

**Figure 2:**
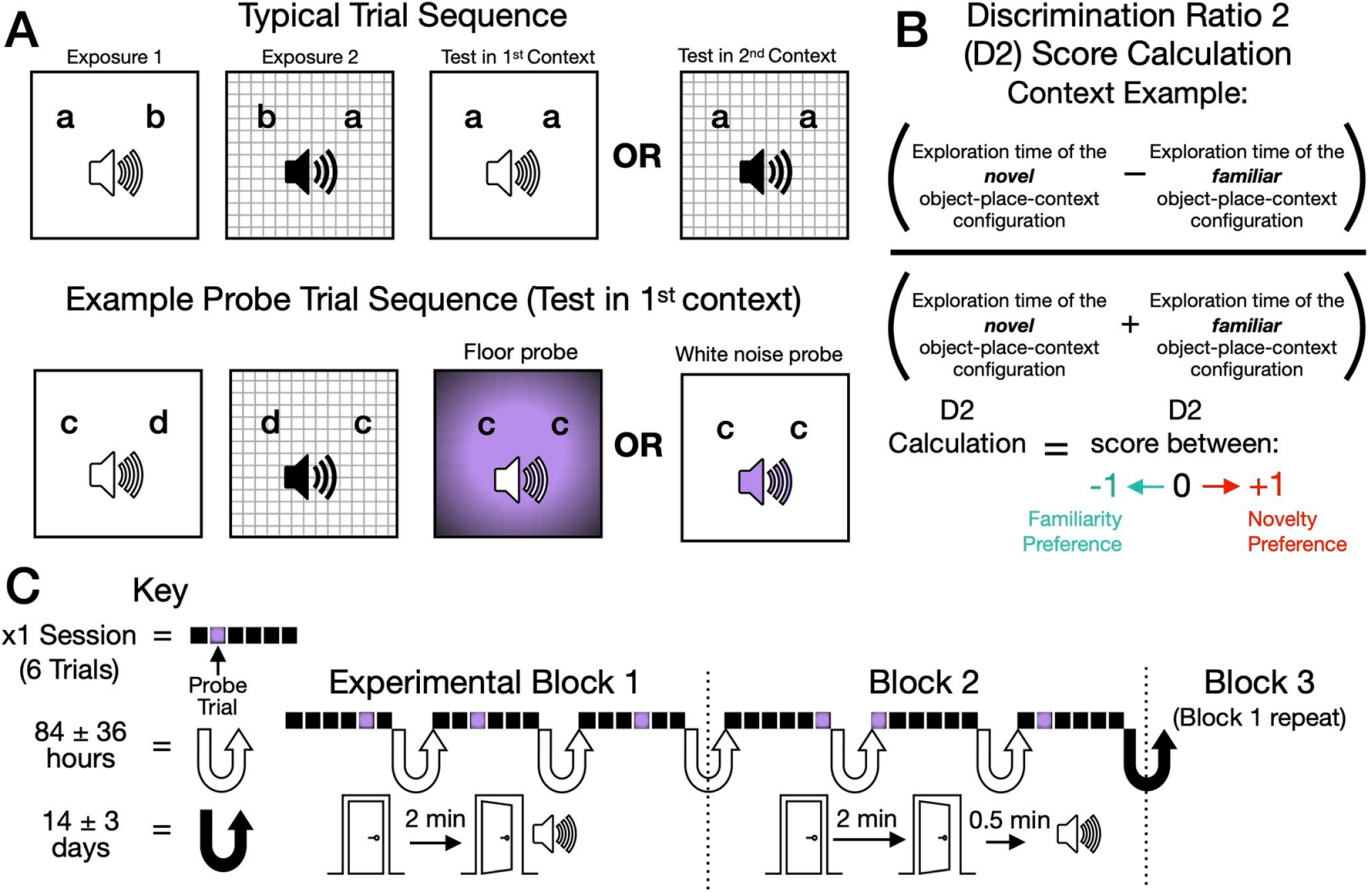
Methodology of trial types and experimental timeline. (**A**) Unexpected object-place-context task. Upper: ‘Contexts’ were comprised of tone-floor pairings. Typical trials: 2 exposure phases (far and middle left; letters denote objects). The test can be made in the 1st context (right-middle) or in the 2nd context (far-right). Exposure and test phases were 2 min, as were the interval between them and the next trial starting in a session. Lower: Example probe trial (test in the 1st context): Floors are replaced (right-middle; tones remain stable) or tones are replaced (far-right; floors remain stable). (**B**) Discrimination ratio 2 (D2) calculation (context example). For each animal, the context D2 score, recency D2 score and side bias was calculated individually for each trial and then averaged across all trials (in a given session/block) and finally across animals to give an average D2 score. We also separated trials occurring before/after probe trials for a given session, and averaged them across a block, creating a before/after probes context and recency average D2 score. This was sometimes separated further by considering the trials only when test was made in the 1st context or only when test was made 2nd context. (**C**) Timeline of the main experimental blocks. Upper: Each block had 3 sessions composed of 6 trials (1 probe per session; the location of which is indicated by the purple square). There were 3 blocks and finally one session where all 6 trials were probe trials (not shown in **C**). We included 1 control probe within each block, where at test there were 2 novel objects present (not previously seen during exposure phases). Block 3 was a within-subject counterbalanced repeat of block 1. Lower: In block 1 and 3 the tonal cue played immediately to the onset of the door opening starting a given exposure/test phase. However, in block 2 we delayed the onset of the tone by 0.5 min, this was relative to the door opening and rats shuttling into the open field chamber of only exposure phases of typical trials.

We used an OPC task where a given context was comprised of both a distinct floor and a unique auditory tone. Importantly, each testing session contained typical trials and a probe trial (Fig. 2; see “Methods”), where at the test phase for the probe we manipulated the previously stable floor-tone context by replacing either the floor, or tone with an unexpected floor or white noise. This created an unexpected ambiguous event as only one of the two contextual elements remained the same. Each experimental block consisted of 3 testing sessions composed of 6 trials (1 probe trial per session). There was a total of 3 experimental blocks and finally one session where all 6 trials were probe trials.

Rats (n = 8) autonomously shuttled through a door separating the holding chamber and open field chamber (where object exploration occurred) until the end of a given session, thus entirely without experimenter handling. Recognition memory performance was evaluated via the discrimination ratio 2 (D2) score^8^, calculated separately for an integrated OPC recognition memory (a context-based strategy; Fig. 2B) or an object-place recency strategy. Both a context and recency D2 score gives a value between − 1 and + 1, where 0 reflects no preference (i.e., chance-level performance, e.g., two-tailed one sample t test). Positive 1 reflects exploration of novelty preference and − 1 reflects exploration of familiarity preference. Side bias calculations controls for if animals were consistently exploring left (+ 1) or right (− 1) objects at test phases.

**Figure 3:**
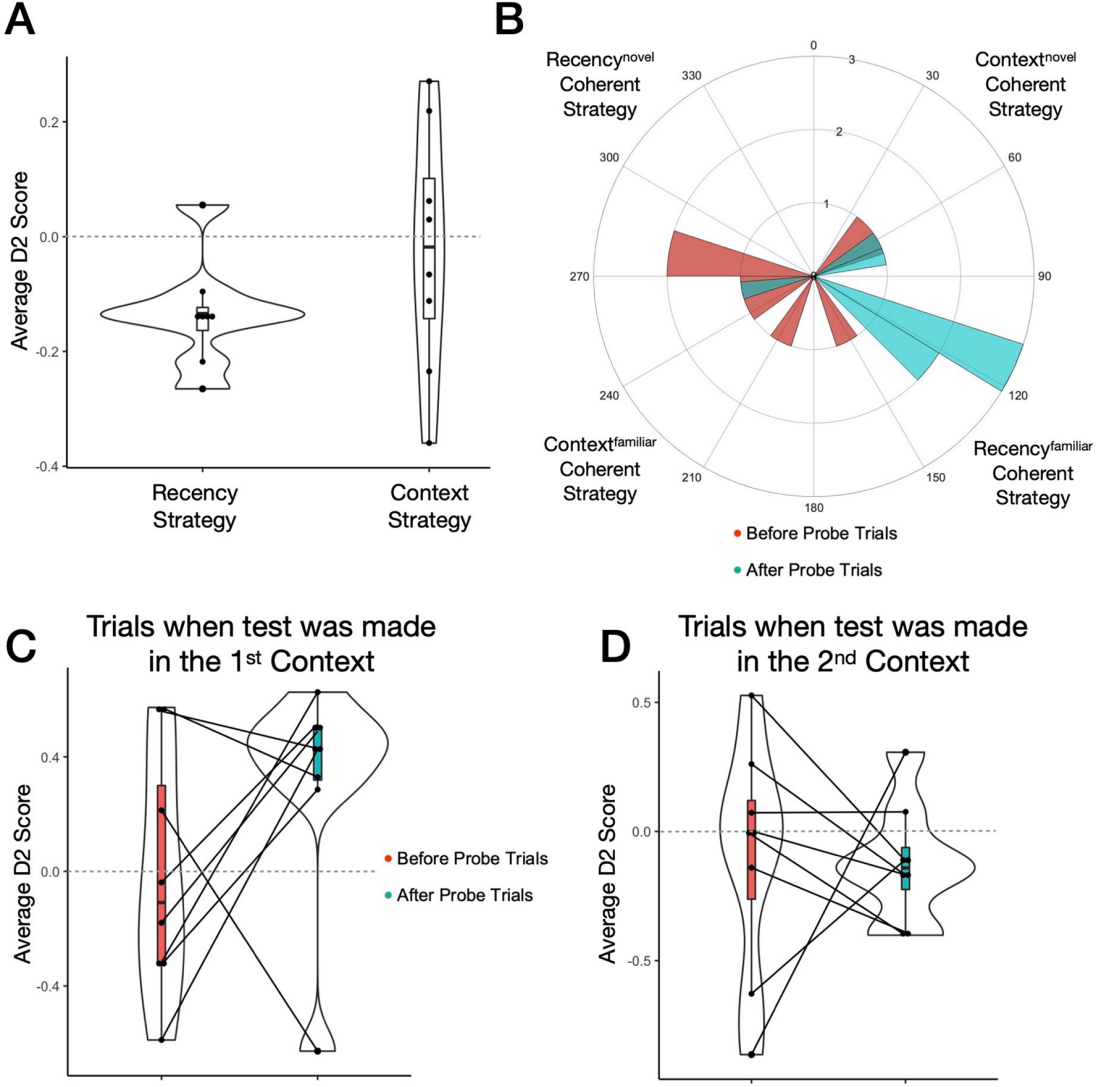
Rats change their behaviour to a familiarity driven recency-based strategy after probe trials. (**A**) Block 1 (probes excluded): The recency D2 (M = − 0.13, SD = 0.09) significantly differed from zero (*t*_(7)_ = − 4.03, *p* = 0.005, *d* = − 1.42, CI 95% − 2.41, − 0.39). The context D2 score (M = − 0.02, SD = 0.22) and side bias (M = − 0.04, SD = 0.17) did not differ from zero (*t*_(7)_ = − 0.31, *p* = 0.76, *d* = − 0.11; *t*_(7)_ = − 0.72, *p* = 0.50, *d* = − 0.25; respectively). (**B**) Angular histogram before probe angles (M = − 109.4°, SD = 85.4°, n = 8), after probe angles (M = 106.0°, SD = 50.2°, n = 8), 20 bins of 18°. The before probe angles were uniformly distributed around the circle (Rayleigh-test: *Z* = 0.87, *p* = 0.43), but the after probe angles were not (Rayleigh-test: *Z* = 3.71, *p* = 0.02). This implies that there was significant directionality to the data after probe trials, in the theoretical direction for a coherent Recency^familiar^ strategy (see Fig. 1F). Also, the before and after probe angles did not have a common mean direction (Watson–Williams F test: *F*_*1,14*_ = 10.40, *p* = 0.006), suggesting a change in behaviour after experiencing probes in block 1. (**C**) Average before/after probes context D2 score from trials only when the test was made in the 1st context (before: M = − 0.01, SD = 0.43; after: M = 0.44, SD = 0.12). The before probes context D2 did not differ from chance (*t*_(7)_ = − 0.09, *p* = 0.93, *d* = − 0.03), however the after probes context D2 score did (*t*_(6)_ = 10.23, *p* < 0.001, *d* = 3.87, CI 95% 1.61, 6.11) and from the before D2 score (*t*_(6)_ = − 2.63, *p* = 0.04, *d* = − 0.99, CI 95% − 1.89, − 0.05). (**D**) Average before/after probes context D2 scores from trials only when test was made in the 2nd context (before: M = − 0.10, SD = 0.45; after: M = − 0.12, SD = 0.23). Both did not differ from zero (*t*_(7)_ = − 0.61, *p* = 0.56, *d* = − 0.22; *t*_(7)_ = − 1.48, *p* = 0.18, *d* = − 0.52; respectively) nor from each other (*t*_(7)_ = 0.11, *p* = 0.92, *d* = 0.04).

In experimental block 1, we first asked whether rats were recognising objects using any strategy more so than chance and initially found that a recency-based strategy exhibited via familiarly preference best explained average recognition performance, across all trials excluding probes (Fig. 3A).

We next asked whether it was the experience of probes that was affecting the strategy that rats used to recognise objects. We observed that before probes neither the average recency D2 score (M = − 0.11, SD = 0.24) nor context D2 (M = − 0.03, SD = 0.39) differed from chance performance (*t*_(7)_ = − 1.35, *p* = 0.22, *d* = − 0.48; *t*_(7)_ = − 0.22, *p* = 0.83, *d* = − 0.08; respectively). In addition, the after probe context D2 also did not differ from chance (M = 0.13, SD = 0.24; *t*_(7)_ = 1.56, *p* = 0.16, *d* = 0.55). However, the recency D2 score after probe trials (M = − 0.27, SD = 0.20) was negative, and significantly differed from chance (*t*_(7)_ = − 3.96, *p* = 0.005, *d* = 1.40, CI 95% − 2.38, − 0.38), with performance being particularly driven from trials when test was made in the 1st context (Fig. 3C). A difference in total exploration did not contribute in explaining the change in behaviour that we observed, as the average total exploration before probes (M = 67.9 s, SD = 38.4 s) did not differ to that after probes (M = 61.6 s, SD = 41.4 s; *t*_(7)_ = 0.48, *p* = 0.65, *d* = 0.17). Therefore, considering these results overall (Fig. 3), there was notable individual variability in performance before probes, whereas after probes, rats seemed to have robustly changed their behaviour to a familiarity driven object in place recency-based strategy on average.

### Rats are capable of a novelty driven context-based recognition strategy in the same task

Unlike experimental blocks 1 and 3, we delayed the onset of the auditory contextual cue by 0.5 min in block 2 testing (Fig. 2C). We hypothesised that this would enhance the salience of contextual cues^18,19^ during these exposure phases and potentially impact strategy implementation.

**Figure 4:**
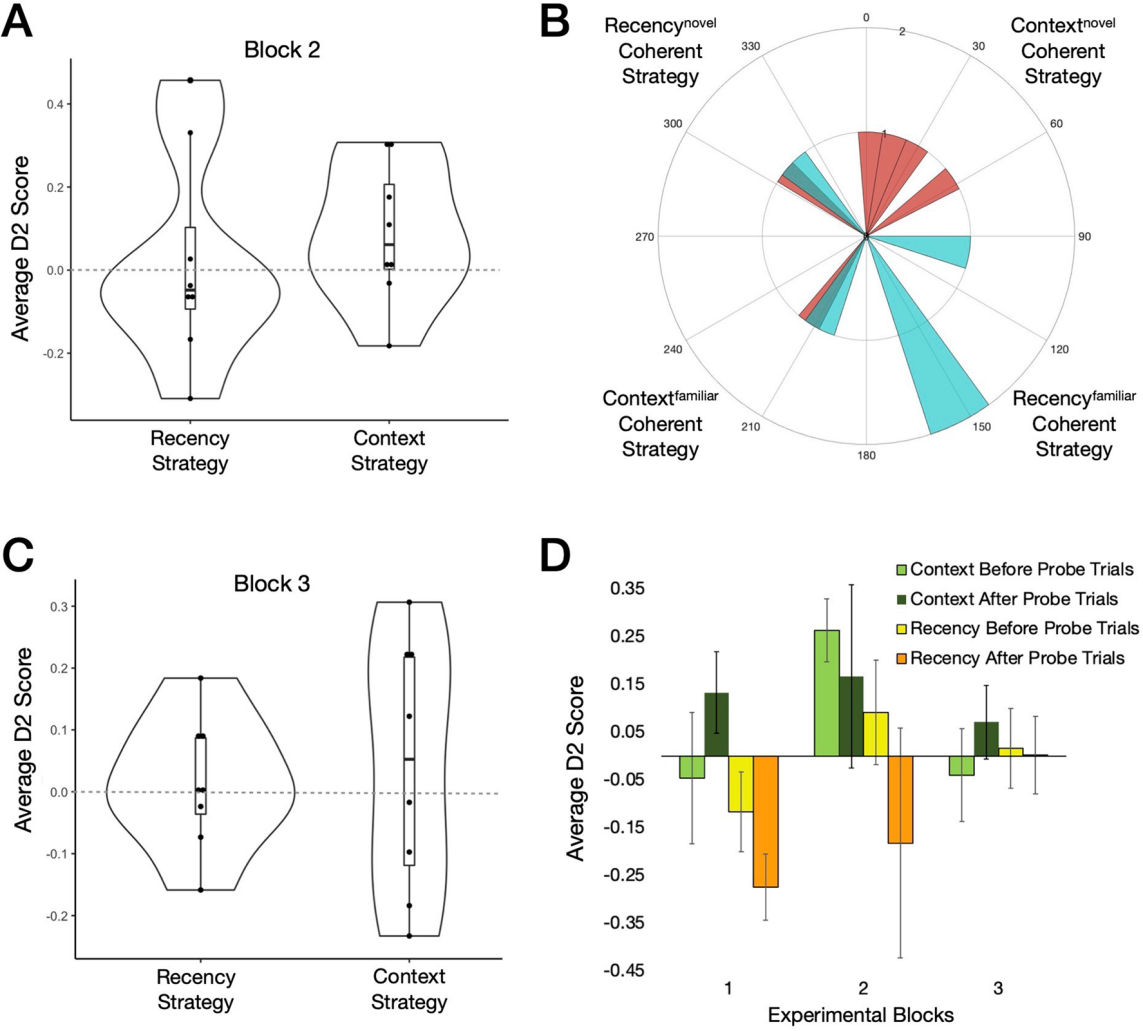
Experimental blocks 2 and 3 and performance across blocks. (**A**) Block 2 (probes excluded): The overall average context D2 score (M = 0.09, SD = 0.17) and the average recency D2 score (M = 0.02, SD = 0.25) did not differ from zero (*t*_(7)_ = 1.47, *p* = 0.18, *d* = 0.52; *t*_(7)_ = 0.24, *p* = 0.82, *d* = 0.09; respectively). There was no side bias present (M = 0.09, SD = 0.24; *t*_(7)_ = 1.01, *p* = 0.35, *d* = 0.36). (**B**) Angular histogram before probe angles (red), after probe angles (blue) for block 2; 20 bins of 18°. Both the before probe trial (M = − 0.2°, SD = 64.8°, n = 6) and the after probe trial angles (M = 159.0°, SD = 74.7°, n = 5) were uniformly distributed around the circle (Rayleigh test: *Z* = 1.67, *p* = 0.19; *Z* = 0.92, *p* = 0.42; respectively). (**C**) Block 3 (probes excluded): The average context D2 score (M = 0.04, SD = 0.20) did not differ from zero (*t*_(7)_ = 0.59, *p* = 0.57, *d* = 0.21), nor did the recency D2 score (M = 0.01, SD = 0.11; *t*_(7)_ = 0.38, *p* = 0.72, *d* = 0.13). Additionally, there was no side bias (M = 0.03, SD = 0.12; *t*_(7)_ = 0.60, *p* = 0.57, *d* = 0.21). (**D**) Average before probe context D2 scores, after probe context D2 scores, before probe recency D2 and after probe recency D2 scores across experimental blocks 1 to 3. Error bars denote ± SEM.

We initially found no evidence of a coherent strategy when considering all trials together excluding probes (Fig. 4A). However, when analysing before versus after probe trials separately, we found that the average context before probe D2 score was positive and significantly differed from chance (M = 0.26, SD = 0.19; *t*_(7)_ = 3.98, *p* = 0.005, *d* = 1.41, CI 95% 0.39, 2.39). The recency before probe D2 also differed significantly although notably to a lesser extent (M = 0.19, SD = 0.17; *t*_(6)_ = 2.91, *p* = 0.027, *d* = 1.10, CI 95% 0.12, 2.04). Moreover, both the after probe average context D2 (M = 0.17, SD = 0.54, n = 8) and recency D2 score (M = − 0.17, SD = 0.68) did not differ from chance (*Z* = 0.34, *p* = 0.74, r = 0.12; *t*_(7)_ = − 0.73, *p* = 0.49, *d* = − 0.26; respectively). The finding that both the before probe context and recency D2 scores were positive and differed from chance, suggested that performance was being driven mainly from trials when test was made in the 2nd context. Indeed, the before probe context D2 from test in the 2nd context trials (M = 0.35, SD = 0.27) was positive, and significantly differed from chance on average (*t*_(6)_ = 3.40, *p* = 0.014, *d* = , 1.30, CI 95% 0.24, 2.31), whereas when only considering trials when test was made in the 1st context, the context D2 before probes did not (M = 0.08, SD = 0.39; *t*_(6)_ = 0.56, *p* = 0.59, *d* = 0.21). Similarly to block 1, the average total time spent exploring before probes (M = 44.3 s, SD = 31.6 s, n = 8) did not differ to that after probes (M = 37.8 s, SD = 49.7 s, n = 8; *Z* = − 0.56, *p* = 0.58, r = − 0.20).

We next asked how the block 2 context and recency D2 scores compared to blocks 1 and 3 before probe trials (Fig. 4D). For the recency D2 score before probe trials, a repeated measures ANOVA revealed a non-significant result with no observed trends (F_2,14_ = 0.98, *p* = 0.40, *η*^2^ = 0.12). On the other hand, the before probes context D2 initially revealed a non-significant result (F_2,14_ = 2.32, *p* = 0.14, *η*^2^ = 0.25), although there was a significant quadratic trend to the data (F_1,7_ = 9.26, *p* = 0.02, *η*^2^ = 0.57), thus we interpreted post-hoc tests. Fisher’s least significant difference post hoc tests revealed no difference between block 1 and block 2 (*p* = 0.10, *d* = 0.67), no difference between block 1 and 3 (*p* = 0.94, *d* = 0.03), but a significant difference between block 2 and block 3 (*p* = 0.041, *d* = 0.88). In consideration of these results overall from block 2 testing (Fig. 4), recognition guided behaviour before probe trials is best explained by a context-based strategy expressed via novelty preference (particularly driven by performance in test in the 2nd context trials). Yet, there was no coherent strategy on average after probe trials in experimental block 2.

### The familiarity driven recency-based recognition strategy after probe trials diminishes over time

Experimental block 3 was a repeat of block 1 conducted 14 ± 3 days after block 2 testing. We found that there was no detectable strategy on average in block 3 trials, when analysing all trials together excluding probes (Fig. 4C). Additionally, there were no strategies present before or after probe trials. Neither the average context D2 score before probes (M = − 0.05, SD = 0.27) and after probes (M = 0.07, SD = 0.22) differed from chance (*t*_(7)_ = − 0.47, p = 0.65; *t*_(7)_ = 0.93, p = 0.39; respectively), nor did the recency D2 before probes (M = 0.02, SD = 0.24) and after probes (M = 0.003, SD = 0.23; *t*_(7)_ = 0.20, p = 0.85; *t*_(7)_ = 0.03, p = 0.97; respectively). There was also no difference in the average total time spent exploring before probes (M = 65.3 s, SD = 37.4 s) versus after probes (M = 75.4 s, SD = 47.2 s; *t*_(6)_ = − 0.57, *p* = 0.59, *d* = − 0.22).

Given that there was a strong after probe trial change in behaviour to a familiarity driven recency-based strategy in block 1 (Fig. 3), we asked how the after probes average recency D2 score compared across blocks (Fig. 4D). A repeated measures ANOVA initially revealed a non-significant result (F_2,14_ = 0.73, *p* = 0.50, *η*^2^ = 0.09), yet there was a significant linear trend to the data (F_1,7_ = 6.31, *p* = 0.04, *η*^2^ = 0.47), so we thus interpreted post-hoc tests. Fisher’s least significant difference post hoc tests revealed no difference between block 1 and block 2 (*p* = 0.73, *d* = 0.13), no difference between block 2 and 3 (*p* = 0.53, *d* = 0.23), but a significant difference between block 1 and block 3 (*p* = 0.04, *d* = 0.89). Thus, there was evidence that the behavioural change of expressing a familiarity driven recency-based strategy, after experiencing probe trials, diminished from block 1 compared to block 3.

### No coherent strategy in probe trials across blocks and in the all probe trial session

**Figure 5:**
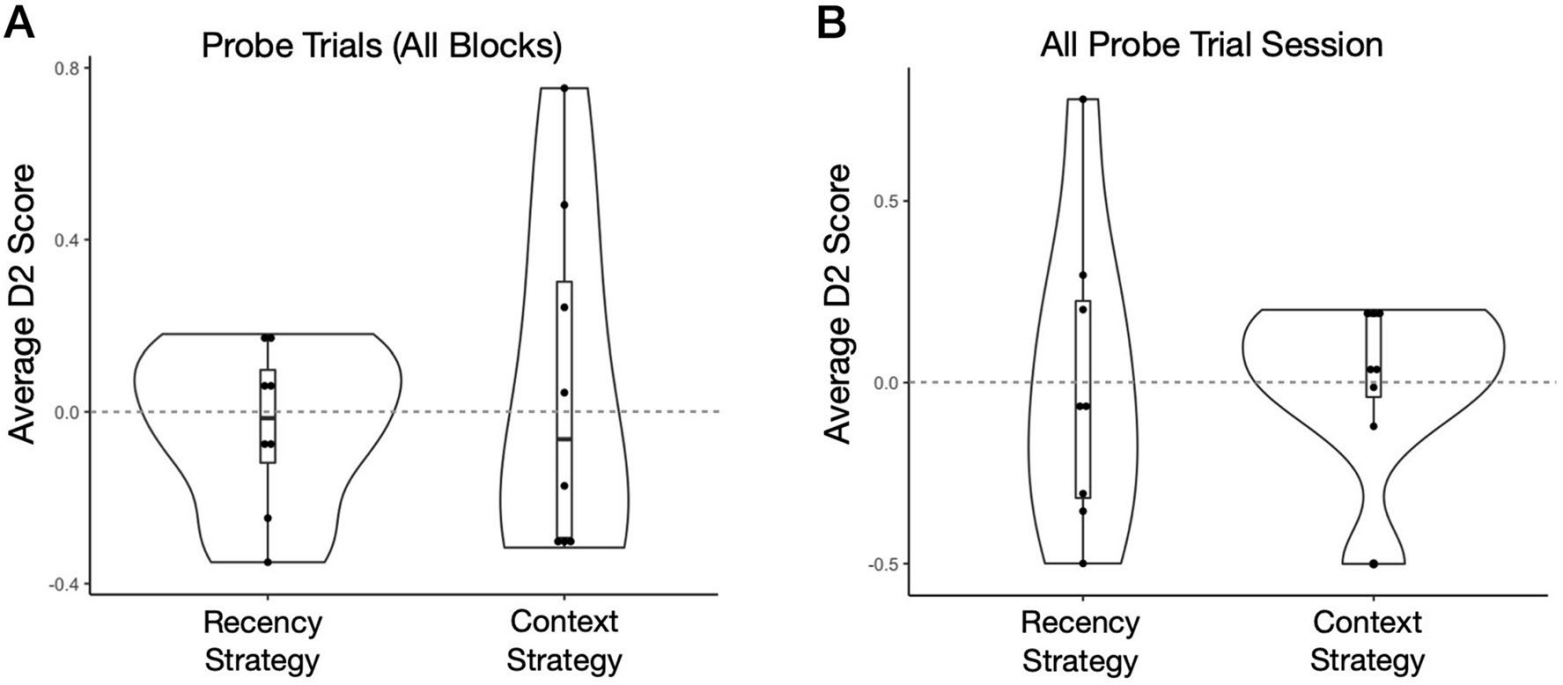
Probe trials across block 1–3 and the all probe trial session. (**A**) Probe trials (averaged across blocks 1–3): The average context D2 score (M = 0.05, SD = 0.40) and recency D2 score (M = − 0.04, SD = 0.20) did not differ from zero (*t*_(7)_ = 0.38, *p* = 0.72, *d* = 0.13; *t*_(7)_ = − 0.53, *p* = 0.61, *d* = − 0.19; respectively). (**B**) All probe trials session: The average context D2 score (M = 0.001, SD = 0.23) and recency D2 score (M = − 0.002, SD = 0.42) did not differ from chance (*t*_(7)_ = 0.01, p = 0.99; *t*_(7)_ = − 0.01, p = 0.99; respectively). Also, there was no side bias present (M = − 0.02, SD = 0.22; *t*_(7)_ = − 0.21, p = 0.84).

There was no clear strategy on average in probe trials averaged across blocks 1–3 nor in the all probe session (Fig. 5). Control probe trials during experimental blocks 1–3, where novel objects were introduced in the test phase not previously seen in exposure phases (one per block), yielded no particular side bias (M = 0.03, SD = 0.40; *t*_(7)_ = 0.19, *p* = 0.86, *d* = 0.07). Moreover, repeated measures ANOVAs revealed no differences or trends between average context probe D2 scores and average recency probe D2 scores across blocks (F_2,14_ = 0.73, *p* = 0.50, *η*^2^ = 0.10; F_2,14_ = 0.68, *p* = 0.52, *η*^2^ = 0.09; respectively). Overall, this suggested that there was great individual variability in recognition behaviour across probe trials, leading to no coherent strategy by rats on average.

## Discussion

For the first time we show that rats change their response in an SOR task based on predictability of the task conditions (Fig. 3). Importantly, no explicit external reinforcement shaped the behaviour. In all cases behaviour was driven by memory of previous events, but the nature of the memory driving the behaviour (recency or episodic) was determined by the predictability of the task conditions.

By manipulating contextual information in unexpected ambiguous events, we posit that this violated the rats’ predictions of the previously stable floor-tone context associations^18–22^, devaluing mnemonic associations reliant on contextual information^34,35^. Thus, the behavioural change after probes to an object in place recency-based recognition strategy (free from a context-based association), allows for maximising mnemonic confidence of past experience^36^, whilst minimising future prediction error^22^ and still behaviourally identifying novelty^1^. Moreover, the familiarity preference of the recency-based strategy that we observed is in accordance with evidence that non-human laboratory animals can express a more conservative approach in their exploratory behaviour^10–12,31,37^. For example, young rats exhibited a developmental switch from familiarity to novelty preference in the novel object in place task^11^.

In the same cohort of rats, we show that they could still exhibit a novelty driven episodic (context-based) strategy on average in the same task (Fig. 4). This is supported by previous findings in other OPC tasks in a continual trials apparatus^24^ and in one trial a day testing^30,38,39^. We postulate that delaying the onset of the tone enhanced the salience of the contextual cues^18,19^, likely recruiting associative mnemonic hippocampal processing^40–47^, as good episodic recognition performance in the OPC task has been seen to be dependent on the hippocampus^38^ and fornix^30^.

As rats progressively experienced a greater number of probe trials, the unexpected nature of them should have lessened (intuitively, becoming more expected over time). There was evidence to support this, as the recency D2 score after probes decreased linearly from block 1 to block 3 (Fig. 4D), suggesting increased variability in their recognition guided behaviour after probes over time. Indeed, in a hippocampal-dependent episodic-like task explicitly using valence, rats could remember episodic integrations over long retentions (> 24 days), but this was similarly accompanied by a notable degree of individual differences in behavioural performance^48,49^. Furthermore, we speculate that the lack of any coherent strategy on average seen during experiential blocks, as well as probe trials themselves, was in part due to individual differences in hippocampal-mediated learning over time^21,43–47^, and especially in block 3, it is possible that rats had retained varying degrees of schematic memory e.g.^50,51^, that ‘probe trials can occur’ in the task. However, future context-SOR work may use probe trials to further explore this possibility.

The reporting of novelty object preference in the literature outweighs that of familiarity preference, notably in relation to context-SOR in rodents^31^. Therefore, there remains many unknowns regarding expression of familiarity preference. For example, in the same group of animals, does the age^11^ and anxiety^12^ factors that influence expression of familiarity preference in one SOR task type (e.g., novel OR) equally influence expression in the novel object-in-place task or a context-SOR task, (despite these various SOR tasks recruiting and relying upon differing neuronal structures^3–6,13–15^)? Moreover, we have used a relatively short time for exposure/test phases and inter-phase-intervals (IPI; 2 min). This may have contributed to the change of recognition behaviour we observed in block 1 and the lack of coherent recognition behaviour at times, as longer exposure phases and IPIs (especially the IPI between exposure 2 and test) have been seen to help stabilise episodic-like memory expressed via novelty preference in one trial a day designs^52,53^. That being said, like in a previous rat continual trials OPC task^24^, which also used short timings (2 min), we similarly observed the emergence of episodic-like memory being expressed via novelty preference. Thus, future work using a continual trials approach to context-SOR may seek to optimise the timings of exposure/test phases and IPIs, depending on the nature of the experiment and practical limitations.

Recognition memory can be modelled as a dual process, where recollective retrieval is a distinct neuro-cognitive process to familiarity-based retrieval^5,54^, (not to be confused with familiarity preference in SOR tasks). There is strong evidence that non-human animals have recollective retrieval capabilities^33,55–57^, which requires mnemonic access to the contextual detail (source information) of the recalled content^4,57^. Interestingly, in an analogous human OPC task, it was found that when using temporal order (recency-based) information to accurately recognise event content, participants could use familiarity or recollection^58^. However, when using source (context-based) information, participants could only rely upon recollection^58^. Our novel analytic approach provides better detection of context and recency-based strategies in relevant SOR tasks (Fig. 1), which allows us to draw greater cross-species parallels to source versus temporal-based in human event memory and perhaps recollective versus familiarity-based retrieval. It also enhances explanatory power of the context-SOR data, where a given manipulation may not be globally impairing recognition memory, but instead could be driving a switch in strategy, which is a necessary issue that future research should consider. Finally, for context-SOR tasks we argue that it should at least become standard practice for experimenters to report discrimination ratio scores both averaged together and separately for trials when test is made in the 1^st^ context, and for trials when test is made in the 2nd context, e.g.^34^.

In conclusion, if behaviour is truly spontaneous and implicit in all object recognition paradigms, it becomes very challenging to interpret why certain strategies do seem to emerge over others, and why behaviour changes after experiencing certain events during these tasks. Based on our findings from these experiments we argue against the spontaneous presumption of behavioural manifestation. We propose that, like any other training-based task, rats are continually learning and are seeking out for information which can ultimately influence their volitional mnemonic-dependant exploratory behaviour. However, in context-SOR tasks these strategies are more easily observed using multiple trials within a session for a single animal (to allow consistent behaviour to be seen), and adopting novel analytic tools which allow the investigation of all possible solutions to the task, not simply experimenter defined novelty.

## Methods

### Subjects

Nine male Lister hooded rats (supplied from Charles River, U.K.) were housed in groups of 3 (aged 5–6 weeks upon arrival; 150 ± 10 g), in a room maintained on a 12-h light–dark cycle (07:00–19:00 h), with daily monitoring of temperature and humidity (20 ± 1 °C; 55 ± 10%; respectively). Each home cage measured 56 × 38 × 22 cm (l × w × h; RC2F, NKP isotec., U.K.) and were equipped with a rat tunnel and a guinea pig shelter (Datesand Limited., U.K.). All stages occurred during the light phase and rats had free availability of food and water ad libitum throughout. Animals were not euthanised as part of the experiments. All experiments were conducted in accordance with the U.K. Animals Scientific Procedures Act (1986) and approved by Durham University AWERB and the Home Office (procedure licence number: PP8877096). Reporting follows the recommendations in the ARRIVE guidelines.

### Apparatus and objects

Rats were tested in an apparatus designed for continual trials rat SOR (Model CI.80514R-1, Campden Instruments., U.K.). The open field was ~ 50 × 50 × 30.5 cm (l × w × h), the holding area was ~ 25.5 × 35 × 30 cm and they were connected via a single doorway ~ 7 × 8 × 9. A pellet dispenser and port (~ 4.5 × 3.5 cm) was present in the holding chamber. Walls and the door were metallic, with red Perspex covering the open field and a transparent Perspex covering the holding area. A speaker and camera were positioned centrally over the open field (~ 50 cm high). The auditory cues were played from a sound generator and were pure tones 1–5 kHz or white noise (62 ± 8.5 dB SPL). In the unexpected object-place-context (OPC) task, 4 ‘contexts’ were comprised of removable, sensorily distinct floors (~ 53 × 50 cm) paired with pure tones, they were as follows: 1 kHz with a cream translucent smooth floor, 2 kHz with a stainless-steel hatched floor, 3 kHz with a red sandpaper floor and 5 kHz with a cream translucent floor with a grid of small holes. For probe trials, a rubber black floor or white noise were used both of which were habituated.

The objects varied in material, shape, size, texture and visual complexion, each object had a minimum of three duplicates and were paired quasi-randomly. Objects were positioned to the far corners of the open field opposite the door (i.e., rats egocentrically had objects left and right to them, as they entered the open field). At the end of testing sessions, objects, floors and the apparatus were cleaned using disinfectant wipes (Clinell^®^ universal wipes, GAMA Healthcare Ltd., U.K.). The scheduling of the camera, door, tones and dispenser operations were controlled automatically by programming (ABET II software; Campden Instruments, U.K).

### Habituation and pretraining

Rats acclimatised to their home room for 10 days before handling. Experimenter handling begun with tunnels first taking place in the home room (~ 10 min per group). Then in cage groups they were transported and handled in the laboratory (dim, diffuse white light from a lamp; 100 W and white noise being played) for ~ 10 min for a further 5 days. The laboratory was where all testing took place.

Briefly, the initial habituation and pretraining were as follows: (1) cage group habituation (30 min), (2) single animal habituation (20 min), (3) shuttle training between the holding area and open field (where animals had to consistently anticipate and or shuttle within the ≤ 2 min abort window) and (4) object habituation in the open field, with pilot OPC trials using only tonal cues as contexts.

Four weeks had elapsed between the last pilot trials and the start of habituation for the unexpected OPC task. Animals were first habituated individually to the stable floor-tone contexts with the door open (~ 15 min), 1 context per day, then 2 contexts per day. During this time, the dispenser delivered a chow pellet (45 mg LabTab™ MLab., Indiana, U.S.) each minute into the port. Next, the shuttle and object habituations occurred together with object exploration in the open field, intervals in the holding area and the abort timer all being 2 min. The dispenser delivered a pellet upon entry to the holding chamber and the experimenter placed a pellet between the objects (equidistant from each object) before object exploration in the open field, motivating shuttling and exploration (i.e., baiting). These pellets were not used as rewards, as they remained consistent throughout exposure/test phases regardless of rats’ object exploration or lack of object exploration (i.e., even when no object exploration occurred). At this stage, all contexts were experienced once (~ 20 min) or twice (~ 40 min), with a different pair of the same objects experienced in each context. This occurred in a single habitation session and it was over 3 consecutive days. Gate errors could occur by an animal not shuttling all the way through the door (e.g., turning back once the door was closing) or not shuttling before the abort timer expired. If 3 gate errors were made within a habituation session, the session was aborted for that day. All objects used during habituation were not used during testing.

### Testing protocol

Eight rats were used in the OPC task, with a continual trials approach^23^ (1 did not learn to shuttle efficiently). Each testing session contained 5 typical OPC trials and 1 probe trial (Fig. 2). Sessions begun by the rat being placed into the holding area and nose poking the pellet port, initiating automatic scheduling. For all trials, each exposure and test phase were timed for 2 min after the animal entered the open field and the door closed. After these 2 min, the animal would then return to the holding area initiating a 2 min interval timer once the door closed and could obtain a pellet dispensed into the port. A 2 min interval remained for the start of the next trial within a given session. This allowed sufficient time for the experimenter to change objects, contexts and bait.

Three testing sessions comprised 1 experimental block and there were 3 main experimental blocks (Fig. 2C). This was followed by an all probe trial session (where each of the 6 trials were probes). The time between each session within a block was 84 ± 36 h. In block 1 and 3, the tonal context played immediately as the door opened to exposure and test phases. However, in block 2, there was 0.5 min delayed onset between the door opening, the animal entering the open field and the tone being played for exposure phases of typical trials (probe trials remained the same as in block 1 and 3). Block 2 testing started 72 ± 24 h after block 1. Block 3 was a within-subject counterbalanced repeat of block 1 and started 14 ± 3 days after block 2. The experiencing of the trial order sequence, context and object order, and placement of the novel OPC integrations were counterbalanced throughout all experiments. Additionally, the position of probe trials were counterbalanced across blocks. Finally, objects were not repeated during blocks 1 and block 2, but were for block 3 as it was a repeat of block 1 and were for the all probe trial session (taken from session 1 of block 2).

### Behavioural analyses

Behaviour was measured off-line via the recorded footage of experimental trials. Object exploratory behaviour was determined as when rats were within ~ 2 cm of the object and actively exploring (i.e., visibly whisking, sniffing or touching it). Actions such as sitting upon the object or using it to support rearing were not considered as exploratory behaviour. The duration of exploration behaviour (s) of each object for a given test phase was manually scored via the ChamberView software (Campden Instruments, U.K.) and were not performed blindly by the experimenter. If animals made gate errors (see habituation and pretraining) within a trial, it was excluded from analyses, and if less than half of all the trials for that session were not completed (i.e., the animal made 3 errors and the session was aborted) the other trials of the session were excluded. However, trial completion rate across the main experimental blocks were comparable with no observed trends (block 1: M = 83.3%, block 2: M = 78.5% and block 3: M = 94.4%; F2,14 = 2.28, *p* = 0.14, *η*^2^ = 0.25). Recognition memory performance was evaluated through the discrimination ratio 2 (D2) score^8^ (Fig. 2B). For integrated OPC recognition memory (a context-based strategy), the context D2 score was calculated as follows: (exploration time of the novel integrated OPC configuration − exploration time of the familiar configuration)/(total exploration time of the novel + familiar configurations). Moreover, an object-place-recency D2 score was also calculated: (exploration time of novel object in place recency − exploration time of the familiar configuration)/(total exploration time of the novel + familiar configurations). Finally, the side bias calculation: (exploration of the left object at test − exploration of the right object at test)/(total exploration of the left and right object). For each animal, the context D2 score, recency D2 score and side bias was calculated individually for each trial and then averaged across all trials (in a given session or block) and finally across animals to give an average D2 score. We also separated trials occurring before/after probe trials for a given session, and averaged them across a block, creating a before/after probes context and recency average D2 score. The D2 score data were tested for normality and a non-parametric alternative was used if *p* was ≤ 0.05, using SPSS (2021, IBM Corp). Outlier cases were identified based on quartiles (where *k* = 2.07)^59^ and were excluded from statistical tests.

One can plot individuals’ test in the 1st context trial average context D2 scores against test in the 2nd context trial average context D2 scores and calculate the angle of given data points (0 ± 180°). If animals are indeed performing differently from chance, angular data allows the use of circular (directional) statistics which can enhance explanatory power^60–62^, in terms of strategy (Fig. 1C–F). We used the MATLAB (2020b, The MathWorks, Inc) circular statistics toolbox^62^, to compute circular descriptive and inferential statistics. Thus, all statistical analyses were performed on the average D2 score or angle across animals and all measures reported were two-tailed tests.

## Supplementary Information

Supplementary Information.

## Data Availability

The data that support the findings of this study are available as part of the supplementary materials or on request from T.W.R., the corresponding author.
